# Dietary Inflammatory Index and risk of nonalcoholic fatty liver disease: nonlinear associations, metabolic mediation, and external validation

**DOI:** 10.3389/fnut.2026.1817870

**Published:** 2026-07-01

**Authors:** Tongyan Chen, Guanghui Zheng, Huiyuan Tan, Lujie Guo, Yang Liu, Xueqing Qian, Shengyu Feng, Yanfen Zhang

**Affiliations:** 1 Laboratory Diagnosis Center, Beijing Tiantan Hospital, Capital Medical University, Beijing, China; 2 Department of Laboratory Medicine, The Second Affiliated Hospital of Harbin Medical University, Harbin, China; 3 Department of Anesthesiology, The Affiliated Hospital of Qingdao University, Qingdao, Shandong, China

**Keywords:** Dietary Inflammatory Index, local cohort validation, mediation analysis, NHANES, nonalcoholic fatty liver disease

## Abstract

**Background:**

Nonalcoholic fatty liver disease (NAFLD) is a common metabolic disorder characterized by chronic low-grade inflammation. The Dietary Inflammatory Index (DII) reflects the inflammatory potential of habitual diet and has been associated with several cardiometabolic diseases. However, the relationship between DII and NAFLD is not fully clarified. In particular, it remains unclear which metabolic factors mediate this association, and whether findings based largely on NHANES data can be confirmed in independent local populations.

**Methods:**

This cross-sectional study included adults from NHANES and an independently collected local cohort. Multivariable logistic regression was used to assess the association between DII and NAFLD. Restricted cubic spline models examined dose–response patterns. Subgroup analyses evaluated the consistency of the findings. Mediation analyses quantified the indirect effects of metabolic factors in the DII–NAFLD association.

**Results:**

In the NHANES analysis, higher DII scores were independently associated with an increased risk of NAFLD after adjustment for demographic, socioeconomic, and clinical covariates. Restricted cubic spline analysis indicated a nonlinear association, with NAFLD risk rising more steeply at higher DII levels. Mediation analyses demonstrated that triglycerides, fasting plasma glucose, and insulin partially mediated the DII–NAFLD relationship, although a significant direct effect of DII persisted. Consistent findings were observed in the independent local cohort, where higher dietary inflammatory scores were also associated with greater NAFLD risk.

**Conclusion:**

DII was positively associated with NAFLD and showed a nonlinear dose–response pattern. This association was partially explained by glucose and lipid metabolic disturbances, and machine learning analysis identified DII as an important predictor of NAFLD. Consistent findings in the local cohort supported the robustness of this association. However, causal relationships cannot be established due to the cross-sectional design.

## Introduction

Nonalcoholic fatty liver disease (NAFLD) has become the most prevalent chronic liver condition worldwide and now constitutes a substantial and growing public health challenge (1, 2). Available epidemiological studies indicate that NAFLD has reached a high global prevalence. It affects approximately one quarter to one third of the adult population. This proportion continues to rise alongside increasing rates of obesity, type 2 diabetes, and metabolic syndrome (3–5). Clinically, NAFLD does not represent a single pathological entity but rather a progressive disease spectrum, spanning from simple hepatic steatosis to nonalcoholic steatohepatitis, advancing fibrosis, cirrhosis, and, in a subset of patients, hepatocellular carcinoma (6–8). Importantly, the impact of NAFLD is not confined to hepatic injury alone. A growing body of evidence indicates that NAFLD is closely intertwined with systemic metabolic dysfunction and is associated with a markedly higher risk of cardiovascular disease and diabetes-related mortality (9–11). In light of the lack of approved pharmacological treatments and the frequently silent presentation of early disease, there is an urgent need to delineate modifiable risk factors and develop effective prevention strategies for NAFLD.

Low-grade inflammation is an important biological mechanism involved in both initiation and progression of NAFLD (12, 13). Hepatic lipid overload induces complex cellular stress processes such as oxidative stress, mitochondrial stress, and endoplasmic reticulum stress and results in pro-inflammatory signaling, and inflammatory cytokines (14, 15). These inflammatory processes in addition to driving hepatocellular damage and fibrotic remodeling are also important in terms of systemic metabolic regulation. It is now apparent that dietary preferences play an important role in determining inflammatory tone and balance (16–18). Rather than individual nutrients, overall dietary patterns better capture the complex and synergistic effects of foods on inflammation (19, 20). These results have been supported by associations with existing circulating inflammatory markers such as C-reactive protein and interleukin-6 (21, 22). Since then the DII is widely used in large-scale population studies to study diet inflammation with respect to other chronic diseases including cardiovascular disease, type 2 diabetes and other diseases (23, 24).

In recent years, an increasing number of epidemiological studies have explored the association between the Dietary Inflammatory Index and NAFLD (25, 26). He et al. reported that higher DII scores were associated with increased odds of NAFLD and provided initial evidence suggesting a potential nonlinear relationship between dietary inflammatory potential and hepatic steatosis (27). Similar associations have also been observed in other population-based studies and cohort analyses, supporting the hypothesis that proinflammatory dietary patterns may contribute to NAFLD development (28, 29). It is interesting to note that the DII has been proven to be significantly correlated with circulating inflammatory and metabolic markers, and there is some evidence that pro-inflammatory diets may also be correlated with glucose and lipid metabolisms, which are the main biology processes governing NAFLD disease (30, 31).

However, most existing studies have focused on overall associations or categorical comparisons. Few have examined whether, and to what extent, metabolic dysregulation mediates the relationship between dietary inflammatory potential and NAFLD. Moreover, current evidence is largely based on NHANES data, with few independent validations. These gaps call for a more comprehensive evaluation using nonlinear modeling and mediation analyses to clarify underlying metabolic pathways. Although the nomenclature has shifted from NAFLD to metabolic dysfunction-associated steatotic liver disease (MASLD), the present analysis retained the NAFLD framework because the NHANES data and existing evidence are based on NAFLD definitions. This also ensures comparability with prior studies, while future research using MASLD criteria is warranted. Therefore, this study aimed to examine the association between DII and NAFLD, assess the mediating roles of key metabolic factors, and validate the findings in an independent local cohort.

## Materials and methods

### Study design and data source

This study was a cross-sectional analysis based on data from the NHANES, a nationally representative survey conducted by the Centers for Disease Control and Prevention (CDC) using a complex, multistage probability sampling design. NHANES collects comprehensive information on demographic characteristics, dietary intake, laboratory measurements, and health-related outcomes of the non-institutionalized U.S. population. The present analysis utilized data from the 2017 to 2020 NHANES cycles. To account for the complex survey design, all baseline descriptions, group comparisons, and regression analyses were performed using appropriate NHANES sample weights, strata, and primary sampling units according to NHANES analytic guidelines. All NHANES study protocols were approved by the National Center for Health Statistics (NCHS) Research Ethics Review Board, and written informed consent was obtained from all participants.

In addition, an independently collected local cohort was included to validate the findings. The local dataset comprised adult participants recruited from the Second Affiliated Hospital of Harbin Medical University. Relevant demographic, anthropometric, and dietary information was collected using standardized questionnaires. The study protocol was approved by the respective institutional ethics committees, and written informed consent was obtained from all participants.

### Study population

Participants aged ≥ 18 years from the NHANES 2017 to 2020 cycles were eligible for inclusion. The initial dataset included 15,560 participants. Participants were excluded if they had missing information on NAFLD status, dietary exposure variables, mediator variables, or key covariates, including education level, poverty–income ratio, smoking status, diabetes status, and hypertension status. After merging the exposure, mediator, outcome, and covariate datasets and applying complete-case criteria, a total of 2,248 participants were included in the final NHANES analysis. The detailed selection process is shown in Supplementary Figure 1.

For the local validation cohort, adult participants with complete dietary and basic demographic information were included. After applying the predefined criteria, 438 individuals were analyzed, including 98 participants with NAFLD and 340 controls.

### Definition of NAFLD

NAFLD was defined based on vibration-controlled transient elastography (VCTE) measurements and alcohol consumption status. Hepatic steatosis was assessed using the controlled attenuation parameter (CAP), measured in the NHANES Mobile Examination Center with the FibroScan^®^ model 502 V2 Touch system (Echosens, Paris, France), equipped with medium (M) or extra-large (XL) probes as appropriate. All examinations were performed by trained and certified health technicians according to standardized NHANES protocols and the manufacturer’s guidelines.

Quality control criteria for valid CAP measurements included at least 10 successful acquisitions, with reliability assessed by the median value, interquartile range (IQR), and the ratio of IQR to the median (IQR/M). An IQR/M ratio < 30% was considered indicative of reliable measurements.

A CAP value ≥ 274 dB/m was used to define hepatic steatosis. Participants were classified as having NAFLD if they had CAP ≥ 274 dB/m and average daily alcohol intake < 30 g/day for men or < 20 g/day for women. Participants with CAP < 274 dB/m and alcohol intake below these thresholds were classified as controls (32).

Alcohol intake was estimated based on 24-h dietary recall data from NHANES by summing ethanol intake from all reported alcoholic beverages. The corresponding dietary variables used for this calculation are detailed in Supplementary Table 1. Sex-specific thresholds for non-excessive alcohol consumption were applied according to established clinical guidelines for NAFLD.

### Assessment of Dietary Inflammatory Index

The DII was calculated to assess the inflammatory potential of participants’ diets, following the standardized methodology developed by Shivappa et al. (29). In this study, 25 dietary components were derived from NHANES 24-h dietary recall data, and the detailed list of components and corresponding NHANES variables is provided in Supplementary Table 1. For each component, individual intake was standardized using the global reference mean and standard deviation to generate a z-score. The z-score was then converted into a centered percentile score, multiplied by the corresponding inflammatory effect score, and summed across all available components to obtain the overall DII. The DII was calculated using absolute dietary intake values without adjustment for total energy intake.

In the local cohort, dietary inflammatory potential was assessed using a food-based Dietary Inflammatory Potential Score (DIPS), rather than the nutrient-based DII used in the NHANES analysis. The DIPS was constructed from predefined food groups classified as pro-inflammatory or anti-inflammatory according to established dietary inflammation frameworks, including the Dietary Inflammatory Index (DII) and empirical dietary inflammatory pattern (EDIP) literature (33, 34). Food groups such as red meat, processed meat, refined grains, fried foods, and sugar-sweetened beverages were categorized as pro-inflammatory, whereas vegetables, fruits, whole grains, legumes, fish, and nuts were considered anti-inflammatory. For each food group, intake frequency and portion size were converted into ordinal intake scores. Component-specific scores were then assigned positive or negative weights (+1 or -1) based on inflammatory direction and summed to generate the overall DIPS.

Unlike the standard DII, which is a nutrient-based index calculated using global reference values and literature-derived inflammatory effect scores, the DIPS is a simplified food-based score adapted to the local dietary questionnaire. It was therefore used as a pragmatic approach to evaluate dietary inflammatory potential in the absence of detailed nutrient-level data.

### Mediation analysis

Based on the logistic regression results, triglycerides, uric acid, fasting plasma glucose, insulin, and alanine aminotransferase were selected as candidate mediators. Each biomarker was examined separately as a continuous mediator using the R package mediation (version 4.5.1). For each single-mediator model, the total effect of DII on NAFLD was decomposed into the average causal mediation effect (ACME), average direct effect (ADE), and total effect, and the proportion mediated was calculated. Confidence intervals were estimated using bootstrap resampling with 150 simulations. All mediation models were adjusted for age, sex, race/ethnicity, education level, poverty–income ratio, smoking status, diabetes, and hypertension. Only single-mediator analyses were conducted; joint or multiple-mediator effects were not assessed in this study.

### Covariates

Covariates were selected a priori based on previous literature and biological relevance. These included age (continuous), sex (male/female), race/ethnicity (Mexican American, other Hispanic, non-Hispanic White, non-Hispanic Black, and other race), education level, poverty income ratio (PIR), smoking status (yes/no), diabetes status (yes/no/borderline), and hypertension status (yes/no).

### Machine learning analysis

Participants were randomly divided into training and validation sets at a ratio of 70:30. Machine learning models were developed using the R package caret (version 7.0.1), with 12 algorithms evaluated under five-fold cross-validation. Hyperparameters were tuned within the cross-validation framework, and the optimal parameter combination was selected according to model discrimination performance. For the random forest model, key hyperparameters, including mtry, were optimized during model training. The included features were DII, age, gender, race, education level, poverty–income ratio, smoking status, diabetes, and hypertension. Model discrimination was assessed by ROC curves. The best-performing model was further interpreted using shapviz (version 0.10.2), with feature importance ranked by SHAP values.

### Statistical analysis

All analyses were performed using R software (version 4.4.1). Continuous variables are presented as mean ± standard deviation, and categorical variables are presented as counts and percentages. Group differences were assessed using Student’s *t*-test or one-way analysis of variance (ANOVA) for normally distributed continuous variables, the Kruskal–Wallis H test for skewed continuous variables, and chi-square tests for categorical variables. Three logistic regression models were constructed: an unadjusted model, a model adjusted for demographic factors, and a fully adjusted model including socioeconomic and lifestyle factors. Results are reported as odds ratios (ORs) with 95% confidence intervals (CIs). A two-sided *P* < 0.05 was considered statistically significant.

## Results

### Baseline characteristics of the study participants

As shown in Table 1, 2,248 participants were included, comprising 840 individuals with NAFLD and 1,408 controls. Compared with controls, participants with NAFLD were older and differed significantly in race/ethnicity and educational level (*P* < 0.001), while no significant differences were observed for sex or smoking status. The NAFLD group exhibited a markedly worse metabolic profile, with higher prevalences of diabetes and hypertension and elevated levels of fasting plasma glucose, triglycerides, alanine aminotransferase, hsCRP, insulin, and uric acid (*P* < 0.001). Total cholesterol, serum creatinine, and AST showed no clear differences between groups. Notably, DII levels were significantly higher among participants with NAFLD, with a greater proportion distributed in higher DII quartiles, indicating a more pro-inflammatory dietary pattern.

**TABLE 1:** Baseline characteristics of participants by non-alcoholic fatty liver disease status.

Variable	Level	Control	NAFLD	*p*
*n*		1,408	840	
Age [mean (SD)]		47.92 (17.46)	53.05 (15.63)	< 0.001
Gender (%)	Female	776 (55.1)	445 (53.0)	0.347
	Male	632 (44.9)	395 (47.0)	
Race (%)	Mexican American	123 (8.7)	148 (17.6)	< 0.001
	Non-Hispanic Black	424 (30.1)	183 (21.8)	
	Non-Hispanic White	495 (35.2)	324 (38.6)	
	Other Hispanic	129 (9.2)	74 (8.8)	
	Other race	237 (16.8)	111 (13.2)	
Education (%)	9–11th grade	152 (10.8)	93 (11.1)	< 0.001
	College graduate or above	398 (28.3)	179 (21.3)	
	High school grad	299 (21.2)	208 (24.8)	
	Less than 9th grade	61 (4.3)	61 (7.3)	
	Some college	498 (35.4)	299 (35.6)	
PIR [mean (SD)]		2.72 (1.65)	2.50 (1.56)	0.002
Smoke (%)	No	830 (58.9)	487 (58.0)	0.683
	Yes	578 (41.1)	353 (42.0)	
Diabetes (%)	Borderline	32 (2.3)	30 (3.6)	< 0.001
	No	1,255 (89.1)	579 (68.9)	
	Yes	121 (8.6)	231 (27.5)	
Hypertension (%)	No	989 (70.2)	419 (49.9)	< 0.001
	Yes	419 (29.8)	421 (50.1)	
DII (%)	Q1	468 (33.2)	94 (11.2)	< 0.001
	Q2	289 (20.5)	273 (32.5)	
	Q3	318 (22.6)	244 (29.0)	
	Q4	333 (23.7)	229 (27.3)	
FPG [mean (SD)]		104.85 (28.01)	127.84 (47.87)	< 0.001
TG [mean (SD)]		86.61 (50.36)	137.67 (95.78)	< 0.001
TC [mean (SD)]		181.76 (39.54)	184.41 (40.61)	0.129
AST [mean (SD)]		20.74 (11.17)	21.67 (10.64)	0.054
ALT [mean (SD)]		18.88 (12.93)	25.48 (17.30)	< 0.001
BUN [mean (SD)]		14.33 (5.06)	15.24 (6.53)	< 0.001
Cr [mean (SD)]		0.90 (0.44)	0.88 (0.47)	0.361
hsCRP [mean (SD)]		3.32 (6.55)	5.41 (7.51)	< 0.001
Insulin [mean (SD)]		10.43 (17.24)	21.52 (28.34)	< 0.001
UA [mean (SD)]		5.17 (1.37)	5.72 (1.43)	< 0.001

Data are presented as mean ± standard deviation and categorical variables as n (%).

### Independent associations of DII and metabolic biomarkers with NAFLD

**FIGURE 1:**
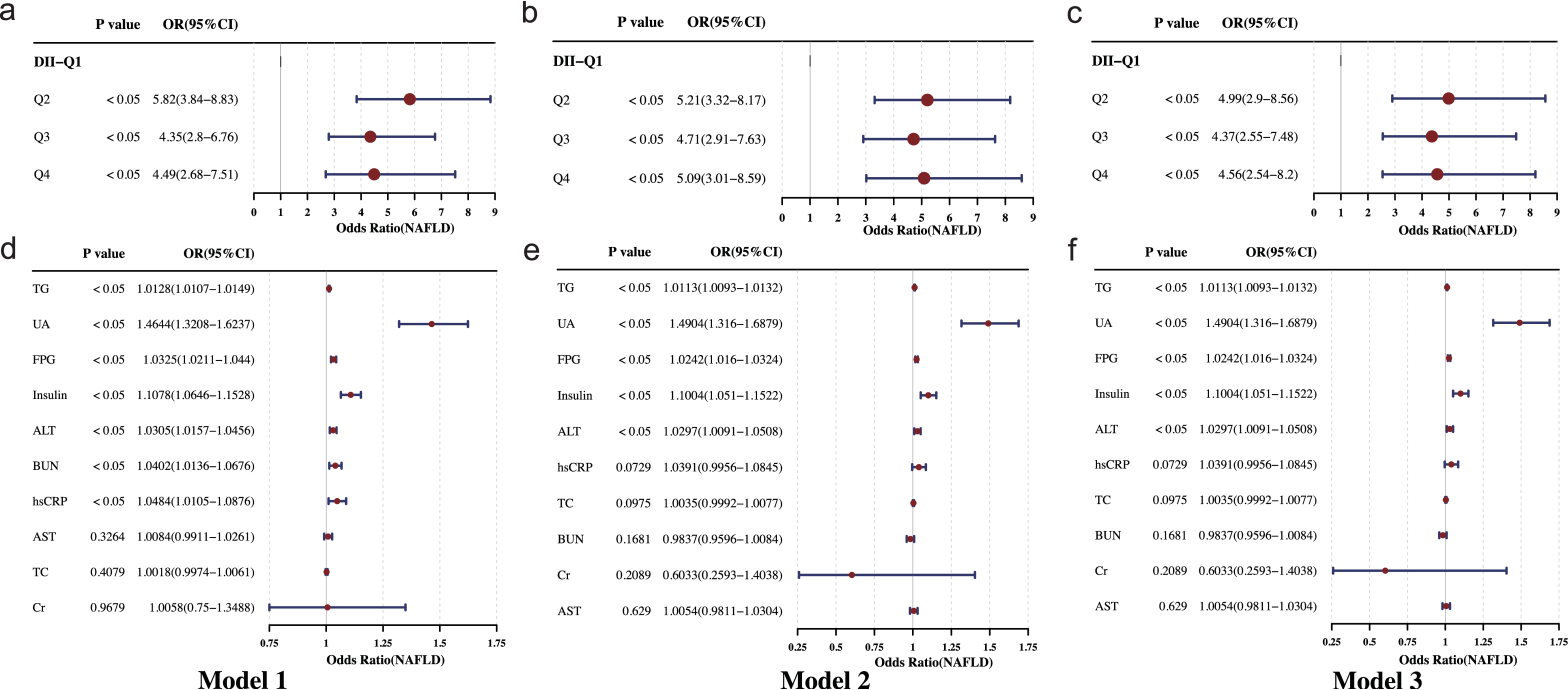
Multivariable associations of Dietary Inflammatory Index and metabolic indicators with NAFLD. **(a–c)** Multivariable logistic regression analyses of the associations between DII quartiles and NAFLD under three models. **(d–f)** Multivariable logistic regression analyses of the associations between metabolic biomarkers and NAFLD under three models (Total sample size: *n* = 2,248). Model 1 was unadjusted; Model 2 was adjusted for age, sex, and race/ethnicity; Model 3 was further adjusted for education level, poverty–income ratio, smoking status, diabetes, and hypertension.

To examine the association between dietary inflammatory potential and NAFLD, multivariable logistic regression analyses were performed using three sequential models (Figure 1), with DII categorized into quartiles and Q1 as the reference. Higher DII quartiles were consistently associated with increased odds of NAFLD across all models, and the association remained stable after adjustment for demographic, socioeconomic, and clinical covariates, indicating an independent and robust relationship between a pro-inflammatory dietary pattern and NAFLD. In addition, higher levels of FPG, TG, insulin, UA, and ALT were associated with increased NAFLD odds, whereas TC, AST, and Cr showed weaker or non-significant associations, providing metabolic context for the subsequent mediation analyses.

### Dose-response relationship between DII and NAFLD

**FIGURE 2:**
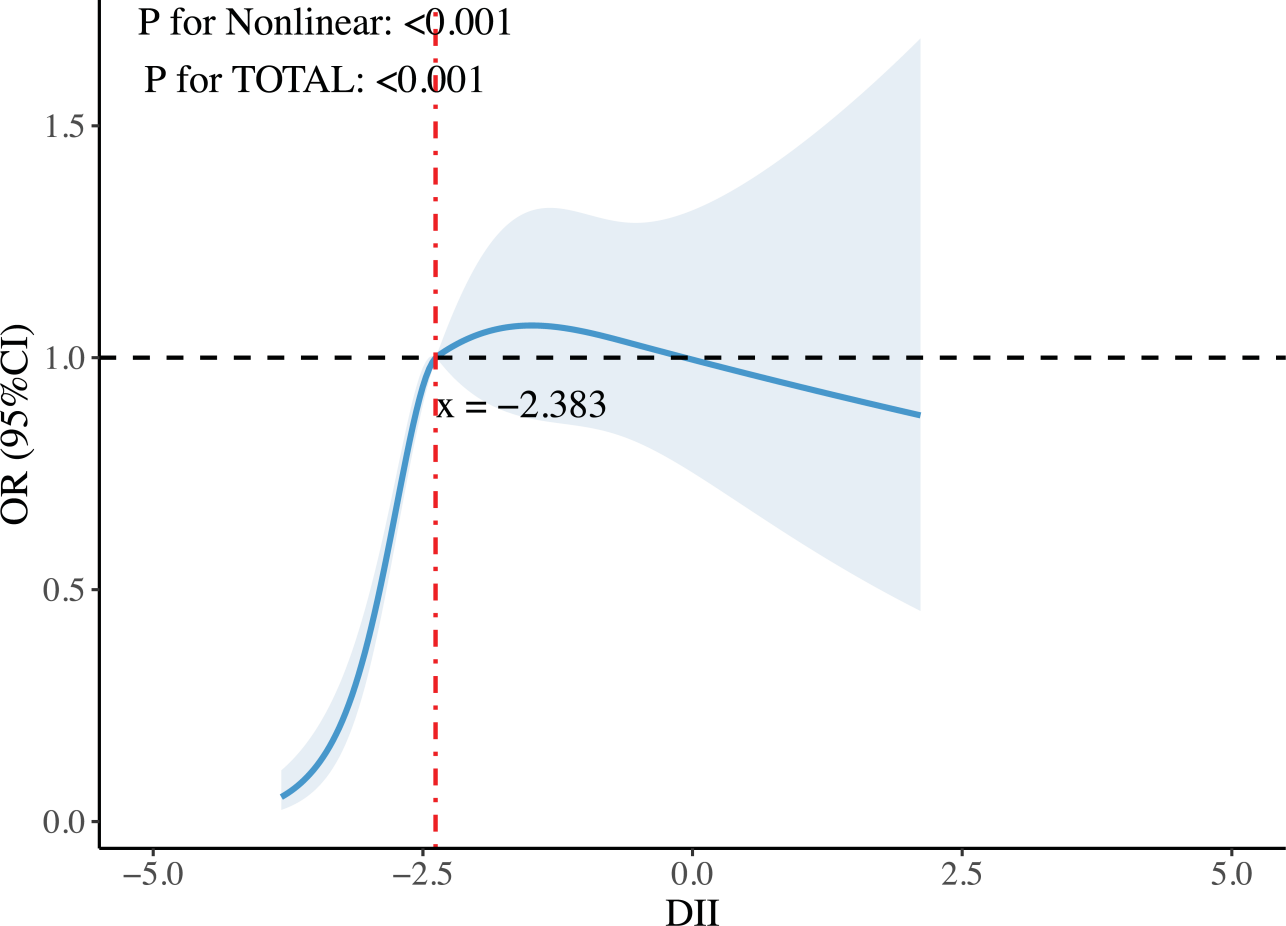
Restricted cubic spline analysis of the association between Dietary Inflammatory Index and NAFLD risk.

To examine the nonlinear association between DII and NAFLD, restricted cubic spline regression was performed (Figure 2). The spline model was fitted with one knot located at DII = −2.383, which was used as the reference point. A significant nonlinear association was observed (*P* for nonlinearity < 0.001), with a strong overall association (*P* < 0.001). The estimated odds of NAFLD remained relatively low at lower DII levels but increased more steeply as DII values rose above the reference point. These findings suggest a nonlinear dose–response pattern, in which higher dietary inflammatory potential is associated with greater NAFLD risk.

The solid blue line represents the estimated odds ratio, and the shaded blue area represents the 95% confidence interval. The horizontal black dashed line indicates the reference risk (OR = 1.00). The vertical red dashed line marks the estimated threshold at which the OR equals 1.00, suggesting a potential nonlinear transition point. *P* for overall and *P* for nonlinearity are shown in the figure. Models were adjusted for age, gender, race, education level, poverty–income ratio, smoking status, diabetes, and hypertension.

### Subgroup analyses

**FIGURE 3:**
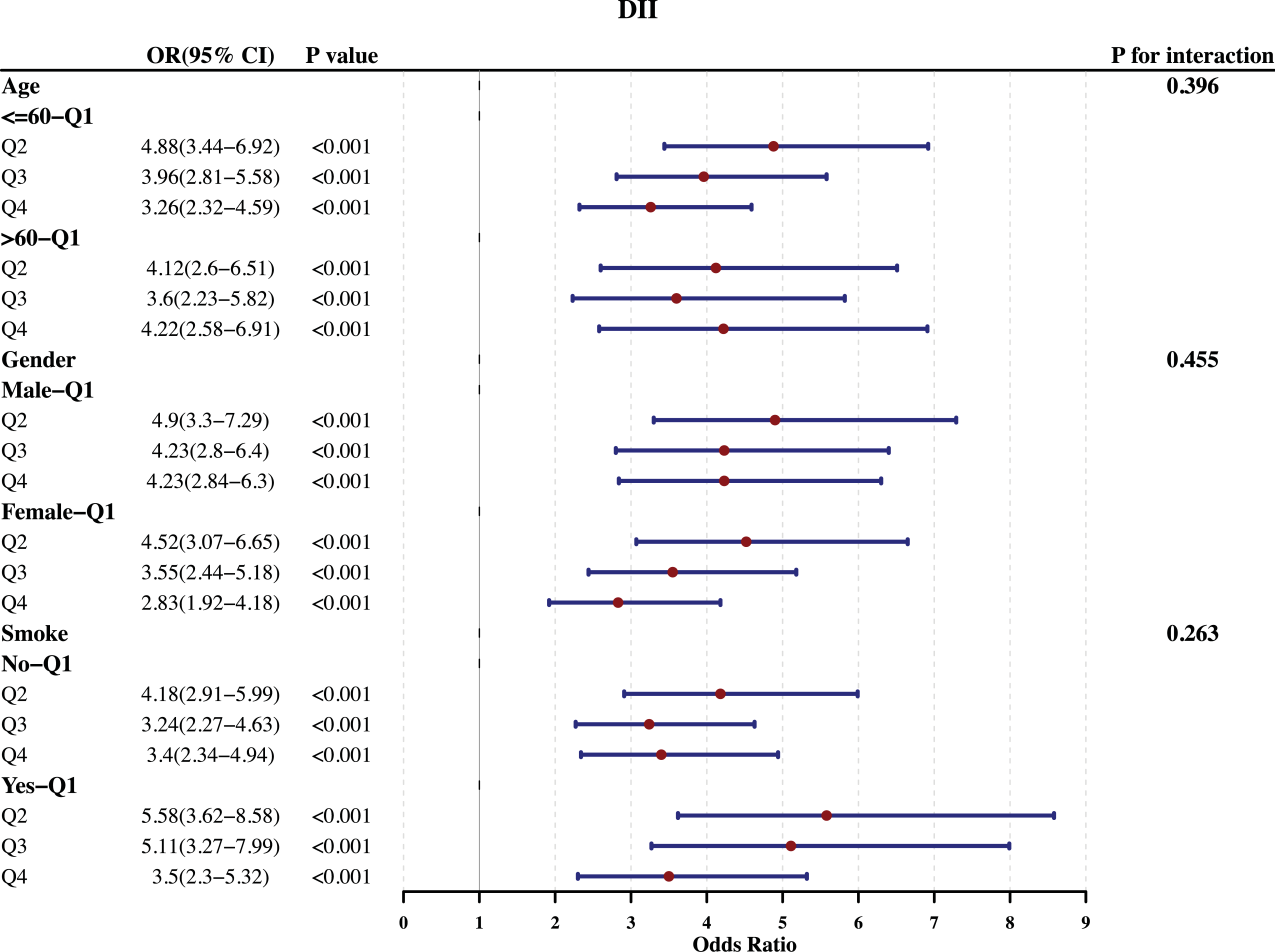
Subgroup analysis of the association between Dietary Inflammatory Index and NAFLD risk stratified by age, gender, and smoking status.

**FIGURE 4:**
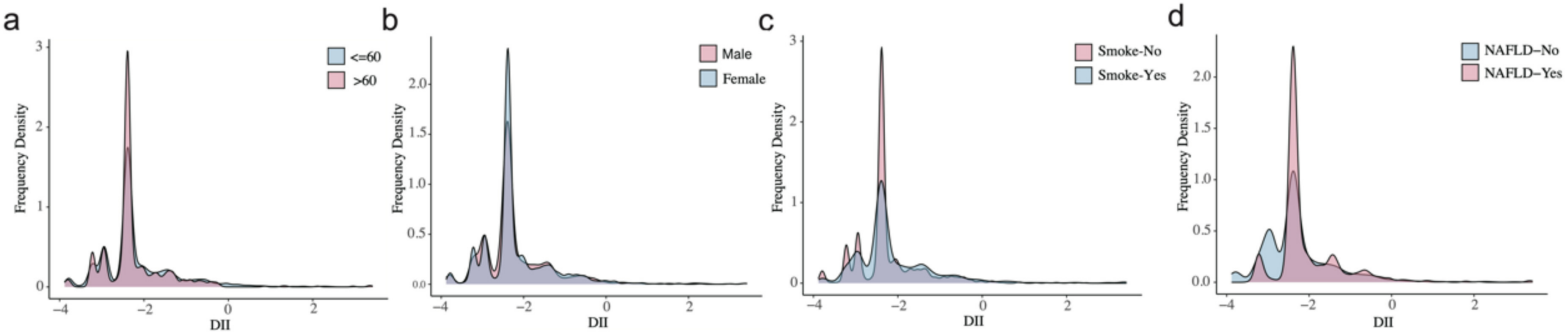
Distribution of Dietary Inflammatory Index scores across population subgroups and NAFLD status. **(a–d)** The distributions of DII scores according to age group, sex, smoking status, and diabetes status, respectively.

Stratified analyses by age, sex, and smoking status were performed to examine potential effect modification (Figure 3). Higher DII levels were consistently associated with increased odds of NAFLD across all subgroups. Although the association appeared slightly stronger among smokers at higher DII levels, no significant interactions were observed for age (*P* for interaction = 0.396), sex (*P* for interaction = 0.455), or smoking status (*P* for interaction = 0.263). Detailed subgroup-specific effect estimates, including sample sizes, odds ratios, and 95% confidence intervals, are presented in Supplementary Table 3. Kernel density plots further showed a right-shifted DII distribution among participants with NAFLD compared with controls across demographic and lifestyle strata (Figure 4), visually supporting the robustness and consistency of the DII–NAFLD association.

The red dots represent odds ratios, and the horizontal blue lines represent 95% confidence intervals. Q1 was used as the reference category in each subgroup analysis. *P* for interaction indicates whether the association between DII and NAFLD differs significantly across subgroup strata. All models were adjusted for age, sex, race/ethnicity, education level, poverty–income ratio, smoking status, diabetes, and hypertension, except for the corresponding stratification variable in each analysis.

### Machine learning performance and SHAP-based interpretability analysis

**FIGURE 5:**
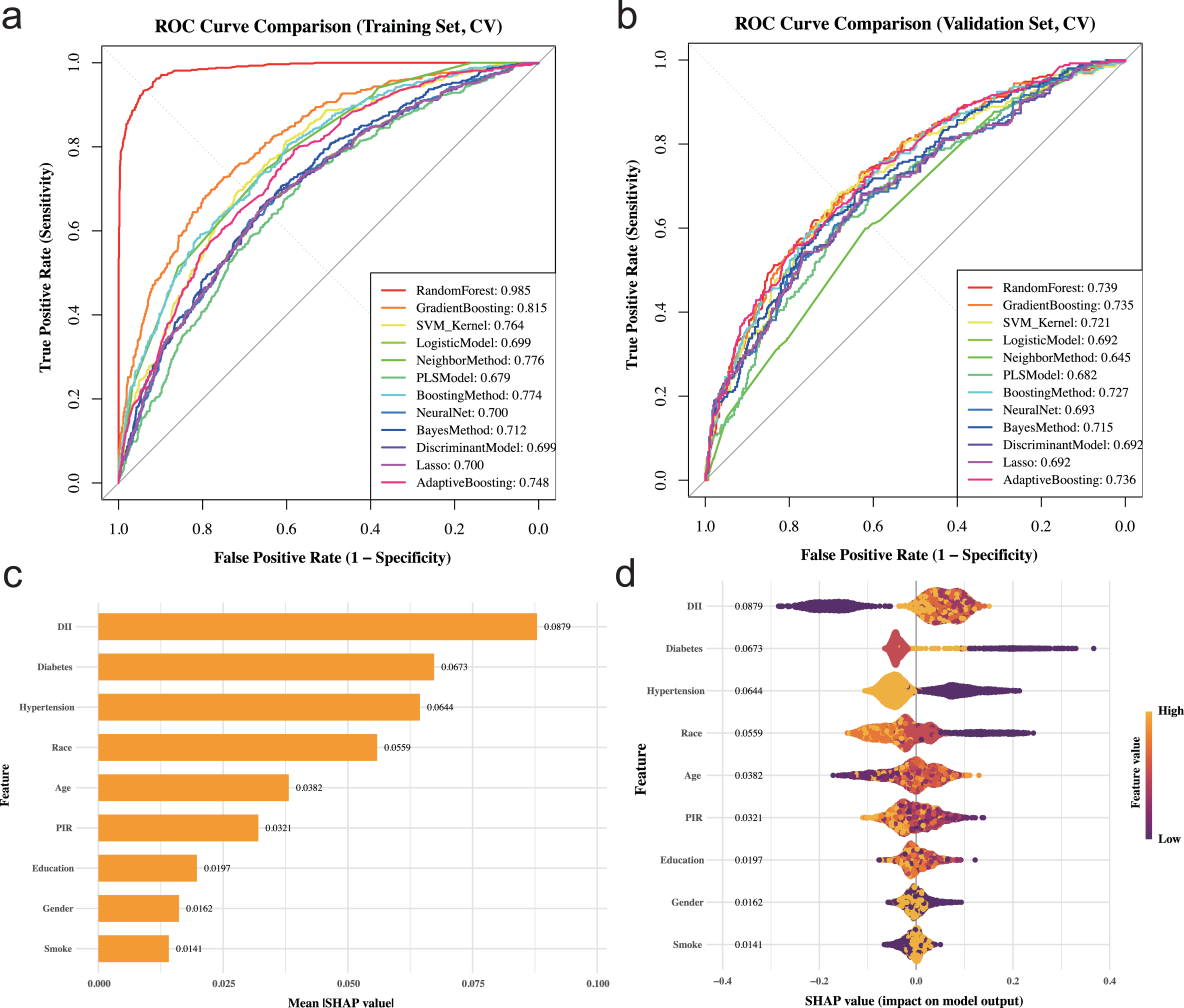
Machine learning performance and SHAP-based interpretability of predictors for NAFLD. **(a)** ROC curves comparing the performance of different machine learning models in the training set using cross-validation. **(b)** ROC curves of the same models in the validation set. **(c)** Feature importance ranked by mean absolute SHAP values from the random forest model. **(d)** SHAP summary plot illustrating the direction and magnitude of each feature’s contribution to model output.

We further constructed machine learning models incorporating DII, age, gender, race, education level, poverty–income ratio, smoking status, diabetes, and hypertension to evaluate their combined performance for NAFLD classification (Figure 5). Most models showed comparable performance, with the random forest model achieving the highest AUC in the training (AUC = 0.985, Figure 5a) and validation sets (AUC = 0.739, Figure 5b). SHAP analyses showed that DII had the largest relative contribution among the included variables (Figures 5c,d). These findings suggest that dietary inflammatory potential may provide additional risk-related information within a multivariable framework, but should not be interpreted as a standalone diagnostic or predictive tool.

### Mediation effects of metabolic factors in the DII–NAFLD association

**FIGURE 6:**
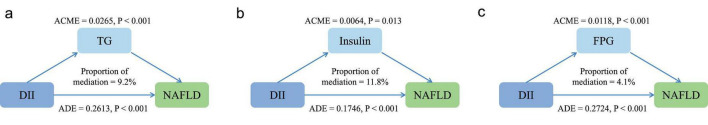
Mediation effects of metabolic factors on the association between Dietary Inflammatory Index and NAFLD. **(a–c)** TG, insulin, and FPG as mediators, respectively. All mediation models were adjusted for age, sex, race/ethnicity, education level, poverty–income ratio, smoking status, diabetes, and hypertension. Solid arrows in the figure indicate statistically significant mediation effects.

To further explore potential pathways linking dietary inflammatory potential to NAFLD, mediation analyses were conducted to quantify the indirect effects of selected metabolic factors (Figure 6). Among the metabolic indicators examined, TG, FPG, and insulin were each evaluated as continuous mediators in the association between DII and NAFLD. The results showed that all three metabolic indicators partially mediated the DII–NAFLD association. Specifically, TG mediated 9.2% of the total effect, while FPG accounted for 4.1% of the association. Insulin also demonstrated a significant mediation effect, explaining 11.8% of the total association. In all analyses, the average direct effects of DII on NAFLD remained statistically significant, indicating that the association was only partially mediated by these metabolic factors.

### Validation of the association between dietary inflammatory potential and NAFLD in a local cohort

**FIGURE 7:**
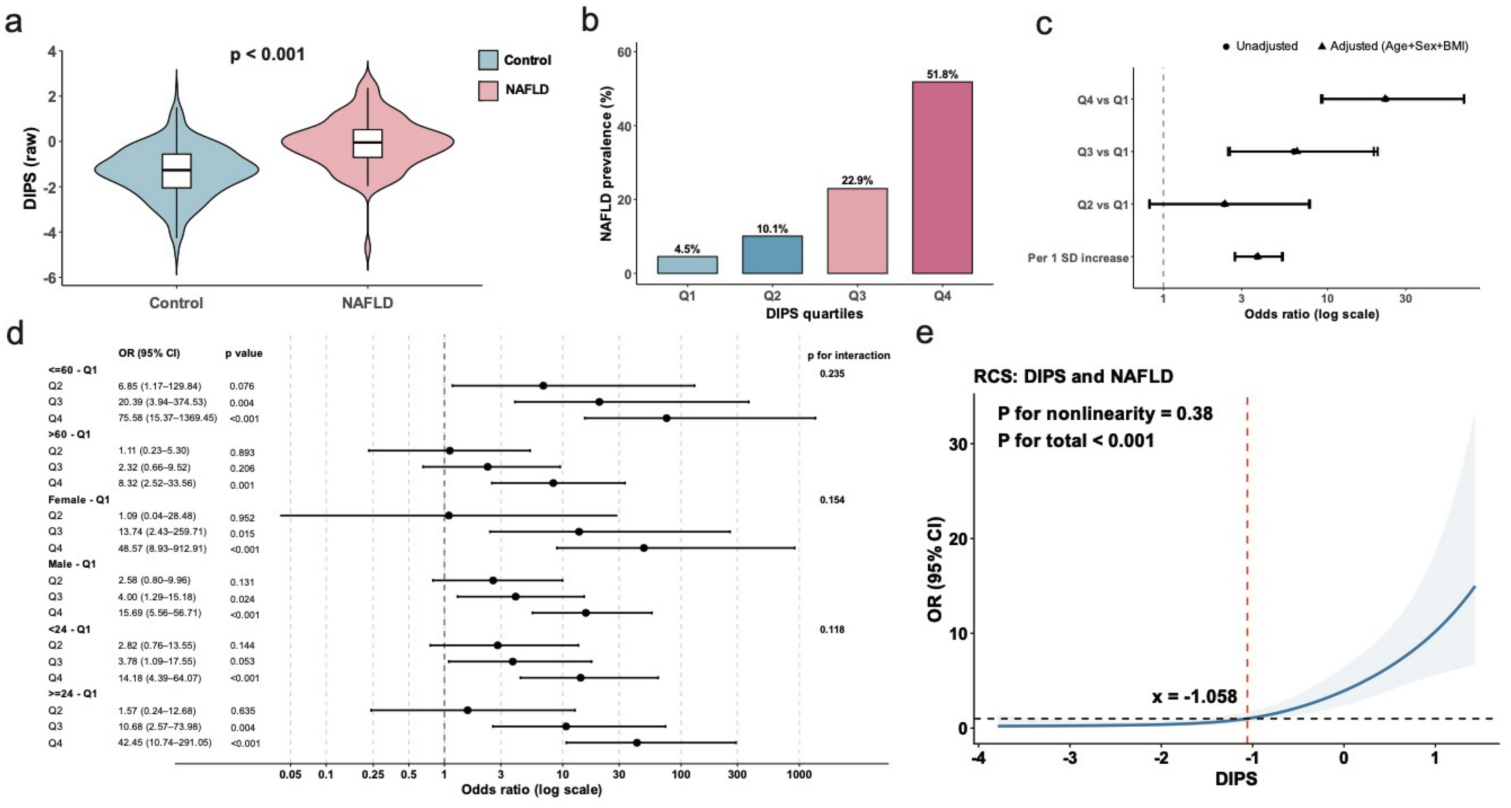
Association between DIPS and NAFLD in the local cohort. **(a)** Distribution of DIPS (raw) values in control and NAFLD groups. **(b)** Prevalence of NAFLD across DIPS quartiles. **(c)** Odds ratios for NAFLD according to DIPS levels (unadjusted and adjusted for age, sex, and BMI). **(d)** Subgroup analyses stratified by age, sex, and BMI **(e)** Restricted cubic spline analysis of the association between DIPS and NAFLD, adjusted for age, sex, and BMI.

In addition to the NHANES analysis, we validated our findings in an independently recruited local cohort. As shown in Table 2, participants with NAFLD were older and had higher body weight than controls, while BMI showed a borderline difference. Importantly, DIPS (raw) values were significantly higher in the NAFLD group, as illustrated in Figure 7a. Quartile analysis further revealed a marked shift toward higher DIPS categories among individuals with NAFLD, with a substantially greater proportion in Q4 and fewer in Q1 (Figure 7b). Consistent with this graded pattern, logistic regression demonstrated progressively increased odds of NAFLD across higher DIPS levels (Figure 7c). Subgroup analyses further showed that this positive association was generally consistent across strata defined by age, sex, and BMI (Figure 7d). No statistically significant interactions were observed (all *P* for interaction > 0.05). Finally, restricted cubic spline analysis confirmed a significant overall association between DIPS and NAFLD, while no evidence of nonlinearity was detected (Figure 7e). The risk of NAFLD increased progressively with higher DIPS values, particularly beyond a threshold region (-1.058). Taken together, these findings mirrored the population-based results and further supported a robust positive association between pro-inflammatory dietary patterns and NAFLD risk.

**TABLE 2:** Association between DIPS and risk of non-alcoholic fatty liver disease in the local cohort.

Variable	Level	Control	NAFLD	*p*
*N*		340	98	< 0.001
Age [mean (SD)]		47.68 (16.21)	53.86 (12.79)	
Gender (%)	Female	164 (48.2)	41 (35.7)	0.263
	Male	176 (51.8)	57 (64.3)	
Height [mean (SD)]		169.98 (8.12)	170.60 (8.34)	0.517
Weight [mean (SD)]		68.02 (9.04)	70.87 (9.12)	0.007
BMI [mean (SD)]		23.70 (3.88)	24.47 (3.51)	0.065
DIPS [mean (SD)]		-1.33 (1.14)	-0.08 (1.12)	< 0.001
DIPS quartiles (%)	Q1	105 (30.9)	5 (5.1)	< 0.001
	Q2	98 (28.8)	11 (11.2)	
	Q3	84 (24.7)	25 (25.5)	
	Q4	53 (15.6)	57 (58.2)	

## Discussion

This nationally representative analysis showed that higher DII scores were associated with an increased risk of NAFLD. The association remained significant after adjustment for demographic, socioeconomic, and metabolic factors. Mediation analyses suggested that triglycerides, fasting plasma glucose, and insulin partially explained the association between dietary inflammatory potential and NAFLD. Machine learning models that incorporated clinical variables further supported these findings, with SHAP analysis identifying DII as the most influential predictor among all variables. Consistent results were also observed in the independent local cohort, supporting the robustness and external validity of the findings. Overall, these results suggest that pro-inflammatory dietary patterns may represent a modifiable factor associated with NAFLD risk.

The association between a pro-inflammatory dietary pattern and NAFLD is biologically plausible and aligns with current understanding of the disease. Higher inflammatory diets may promote systemic low-grade inflammation. This may further aggravate insulin resistance, lipid metabolism disorders, and hepatic steatosis (35, 36). Chronic inflammatory signaling may further increase oxidative stress and mitochondrial dysfunction in hepatocytes, thus promoting the transition of simple steatosis to more severe stages of the disease (37, 38). In addition, the gut–liver axis may play an important role in metabolic liver diseases. Pro-inflammatory diets have been linked to changes in gut microbiota composition, such as reduced microbial diversity and increased pro-inflammatory taxa (20, 39). These changes may impair the intestinal barrier and increase intestinal permeability (40, 41). As a result, bacterial products such as lipopolysaccharide (LPS) may enter the portal circulation more easily. LPS can activate hepatic Kupffer cells and stimulate the release of inflammatory cytokines, including tumor necrosis factor-α and interleukin-6 (42, 43). These inflammatory responses have been associated with hepatic inflammation and steatosis (20, 44, 45). These pathways provide an integrated framework for understanding how diet-related inflammation may linked to NAFLD. They also support the concept that habitual diet can influence liver metabolic health through systemic inflammatory and metabolic effects.

To further clarify the observed association between DII and NAFLD, We conducted mediation analyses. Triglycerides, fasting plasma glucose, and insulin were each examined as potential mediators. All three showed moderate but statistically significant mediation effects. These findings suggest that metabolic factors related to glucose and lipid metabolism may partially account for the association between a pro-inflammatory diet and NAFLD. These findings are consistent with the close associations of insulin resistance and dyslipidemia with NAFLD. In addition, dietary inflammatory patterns have been linked to systemic metabolic alterations, which may be reflected in changes in circulating metabolic markers such as TG, FPG, and insulin (46, 47). More importantly, there was still a large portion of the direct effect of DII after mediating through these mediators, suggesting that the dysregulation of metabolism alone cannot fully explain this association. This residual association may reflect the involvement of other unmeasured factors related to inflammation or metabolic regulation. Overall, the findings support the notion that the relationship between dietary inflammatory potential and NAFLD is complex and likely influenced by multiple correlated physiological processes, rather than being explained by a single pathway.

A major finding of this study is the nonlinear association between DII and NAFLD risk. The risk of NAFLD did not increase uniformly across the full range of DII values, but rose more steeply at higher levels of dietary inflammatory potential. In the present analysis, a turning point was observed at approximately -2.383, beyond which the odds of NAFLD increased more markedly. This pattern suggests a potential threshold-like relationship. However, this threshold should be interpreted as an exploratory statistical finding rather than a clinically validated cut-off value. Biologically, this pattern may reflect reduced metabolic adaptability under higher inflammatory and metabolic burden. Such conditions have been associated with increased hepatic lipid accumulation and liver injury (48). In terms of public health, such a nonlinearity implies that relatively small reductions in the dietary inflammatory potential of people with high DII could result in relatively large reductions in NAFLD risk (49, 50).

From a clinical and public health perspective, these findings are important. Dietary inflammatory potential appears to be a modifiable risk factor for NAFLD. Reducing the inflammatory capacity of habitual diet may help lower the prevalence of NAFLD. The Dietary Inflammatory Index is not a tool for clinical diagnosis. However, it can help identify individuals whose diets promote inflammation. These individuals may benefit from lifestyle changes aimed at reducing dietary inflammation. The observed nonlinear association suggests that individuals with higher DII values may represent a high-risk group. Improvements in dietary quality in this group may be associated with a greater reduction in NAFLD risk. At the population level, these results support more widespread public health strategies that advocate for anti-inflammatory dietary patterns as part of all kinds of metabolic disease prevention. It is important to note that the use of DII in this case is not intended to replace the existing clinical risk factors, but to complement the existing clinical risk factors to provide additional dimensions for risk communication and prevention.

In the local validation cohort, higher DIPS values were consistently associated with increased NAFLD risk, reinforcing the findings from the NHANES analysis. Although the calculation of DIPS is less standardized and less comprehensive than the nutrient-based DII, it reflects real-world food consumption patterns more directly. As DIPS is constructed from food groups, it provides more direct clinical and public health implications. Higher scores were generally characterized by greater intake of pro-inflammatory foods, such as red and processed meats, refined grains, and sugar-sweetened beverages, which have been associated with elevated levels of inflammatory markers including CRP and IL-6 (51). In contrast, lower DIPS values were generally driven by higher consumption of anti-inflammatory foods, including vegetables, fruits, whole grains, fish, and nuts. These foods are rich in fiber, antioxidants, and unsaturated fatty acids. They have been linked to reduced systemic inflammation and improved metabolic profiles (52, 53). These observations suggest that shifting overall dietary patterns toward anti-inflammatory food choices may be a feasible strategy to lower dietary inflammatory burden and reduce NAFLD risk. Notably, the NHANES cohort represents a nationally representative U.S. population, whereas the local validation cohort was derived from two medical centers in northern China, with a smaller sample size and different demographic and clinical characteristics. Despite these differences, the consistent direction of associations across the two cohorts supports the robustness of the findings. However, the applicability of the local validation results may be more relevant to similar northern Chinese populations, and further validation in larger and more diverse cohorts is warranted.

Several limitations should be considered when interpreting the present findings. First, due to the cross-sectional design, causal inference cannot be established, and the temporal relationship between dietary inflammatory potential and NAFLD remains unclear. Second, dietary intake was assessed using 24-h dietary recall, which is subject to recall bias and day-to-day variation, and may not accurately reflect long-term habitual dietary patterns. This may lead to misclassification of DII, and such non-differential measurement error is likely to attenuate the observed associations. Third, although we adjusted for a range of demographic, socioeconomic, and metabolic covariates, residual confounding cannot be fully excluded. Important factors such as total energy intake, overall dietary quality, physical activity, sleep duration and family history of fatty liver disease were not fully accounted for. These variables are known to be associated with both dietary patterns and metabolic health and may have influenced the observed associations. Fourth, potential selection bias should also be considered. Participants with missing data were excluded from the analysis, which may have resulted in a study population that differs systematically from the target population. In addition, DII and DIPS are not clinical diagnostic tools for NAFLD. They should be interpreted as dietary inflammatory indicators for epidemiological risk assessment rather than diagnostic biomarkers. Finally, the mediation analyses were conducted in an observational setting and should be interpreted with caution. The identified mediators represent statistical associations rather than confirmed biological pathways, and causal mechanisms cannot be established.

## Conclusion

In conclusion, while prior studies have reported associations between DII and NAFLD, our study extends the existing literature in several important ways. First, we applied flexible modeling strategies to demonstrate a nonlinear dose–response relationship between dietary inflammatory potential and NAFLD risk. Second, mediation analyses identified triglycerides, fasting plasma glucose, and insulin as statistical mediators, suggesting that glucose and lipid metabolic disturbances may partly explain the association between pro-inflammatory diets and NAFLD. Third, machine learning models integrating DII with demographic and metabolic factors further highlighted the relative importance of dietary inflammatory potential in risk prediction. Finally, the consistency of findings in an independently collected local cohort strengthens the external validity of our results.

## Data Availability

The original contributions presented in this study are included in the article/Supplementary material, further inquiries can be directed to the corresponding authors.
