# Supplementary material for: Dietary Inflammatory Index and risk of nonalcoholic fatty liver disease: nonlinear associations, metabolic mediation, and external validation
Source: Front Nutr. 2026 Jul 1;13:1817870. doi: 10.3389/fnut.2026.1817870 (PMC13368503; doi:10.3389/fnut.2026.1817870)
Supplement: Supplementary file 2 [file Table_1.DOCX]

**Table S1**. Components and parameters used for the calculation of the Dietary Inflammatory Index

| **Covariates** | **Nhanes** | **Abbreviation** | **Overall inflammatory effect scorec** | **Global daily mean intake (units/d)** | **Standard deviation of the global daily intake** |
| --- | --- | --- | --- | --- | --- |
| Alcohol (gm) | DR1IALCO  DR2IALCO | ALCO | -0.277 | 13.98 | 3.72 |
| Vitamin B12(mcg) | DR1IVB12  DR2IVB12 | VB12 | 0.106 | 5.15 | 2.7 |
| Vitamin B6 (mg) | DR1IVB6  DR2IVB6 | VB6 | -0.365 | 1.47 | 0.74 |
| Beta-carotene (mcg) | DR1IBCAR  DR2IBCAR | BCAR | -0.584 | 3718 | 1720 |
| Caffeine (mg) | DR1ICAFF  DR2ICAFF | CAFF | -0.11 | 8.05 | 6.67 |
| Carbohydrate (gm) | DR1ICARB  DR2ICARB | CARB | 0.097 | 272.2 | 40 |
| Cholesterol (mg) | DR1ICHOL  DR2ICHOL | CHOL | 0.11 | 279.4 | 51.2 |
| Energy (kcal) | DR1IKCAL  DR2IKCAL | KCAL | 0.18 | 2056 | 338 |
| Total fat (gm) | DR1ITFAT  DR2ITFAT | TFAT | 0.298 | 71.4 | 19.4 |
| Dietary fiber (gm) | DR1IFIBE  DR2IFIBE | FIBE | -0.663 | 18.8 | 4.9 |
| Folic acid (mcg) | DR1IFA  DR2IFA | FA | -0.19 | 273 | 70.7 |
| Iron (mg) | DR1IIRON  DR2IIRON | IRON | 0.032 | 13.35 | 3.71 |
| Magnesium (mg) | DR1IMAGN  DR2IMAGN | MAGN | -0.484 | 310.1 | 139.4 |
| Total monounsaturated fatty acids (gm) | DR1IMFAT  DR2IMFAT | MFAT | -0.009 | 27 | 6.1 |
| Niacin (mg) | DR1INIAC  DR2INIAC | NIAC | -0.246 | 25.9 | 11.77 |
| Protein (gm) | DR1IPROT  DR2IPROT | PROT | 0.021 | 79.4 | 13.9 |
| Total polyunsaturated fatty acids (gm) | DR1IPFAT  DR2IPFAT | PFAT | -0.337 | 13.88 | 3.76 |
| Vitamin B2 (mg) | DR1IVB2  DR2IVB2 | VB2 | -0.068 | 1.7 | 0.79 |
| Total saturated fatty acids (gm) | DR1ISFAT  DR2ISFAT | SFAT | 0.373 | 28.6 | 8 |
| Selenium (mcg) | DR1ISELE  DR2ISELE | SELE | -0.191 | 67 | 25.1 |
| Vitamin B1 (mg) | DR1IVB1  DR2IVB1 | VB1 | -0.098 | 1.7 | 0.66 |
| Vitamin A, RAE (mcg) | DR1IVARA  DR2IVARA | VARA | -0.401 | 983.9 | 518.6 |
| Vitamin C (mg) | DR1IVC  DR2IVC | VC | -0.424 | 118.2 | 43.46 |
| Vitamin E (mg) | DR1IATOA  DR2IATOA | ATOA | -0.419 | 8.73 | 1.49 |
| Zinc (mg) | DR1IZINC  DR2IZINC | ZINC | -0.313 | 9.84 | 2.19 |

**Table S2**. Performance of machine learning models for predicting NAFLD in the training and test sets

|  | **Train set** | | | **Test set** | | |
| --- | --- | --- | --- | --- | --- | --- |
| **Method** | **AUC** | **X95_CI_Lower** | **X95_CI_Upper** | **AUC** | **X95_CI_Lower** | **X95_CI_Upper** |
| AdaptiveBoosting | 0.748 | 0.724 | 0.772 | 0.736 | 0.697 | 0.774 |
| Lasso | 0.7 | 0.673 | 0.726 | 0.692 | 0.65 | 0.733 |
| DiscriminantModel | 0.699 | 0.673 | 0.726 | 0.692 | 0.65 | 0.733 |
| BayesMethod | 0.712 | 0.686 | 0.738 | 0.715 | 0.675 | 0.755 |
| NeuralNet | 0.7 | 0.673 | 0.726 | 0.693 | 0.652 | 0.734 |
| BoostingMethod | 0.774 | 0.751 | 0.798 | 0.727 | 0.688 | 0.767 |
| PLSModel | 0.679 | 0.652 | 0.706 | 0.682 | 0.641 | 0.723 |
| NeighborMethod | 0.776 | 0.753 | 0.798 | 0.645 | 0.604 | 0.687 |
| LogisticModel | 0.699 | 0.673 | 0.726 | 0.692 | 0.65 | 0.733 |
| SVM_Kernel | 0.764 | 0.741 | 0.788 | 0.721 | 0.682 | 0.761 |
| GradientBoosting | 0.815 | 0.793 | 0.836 | 0.735 | 0.696 | 0.774 |
| RandomForest | 0.985 | 0.981 | 0.99 | 0.739 | 0.701 | 0.778 |

**Table S3**. Subgroup analyses of the association between DII and NAFLD

| **Variable** | **Count** | **Percent** | **Levels** | **OR** | **Lower** | **Upper** | **P value** | **P for interaction** |
| --- | --- | --- | --- | --- | --- | --- | --- | --- |
| Overall | 2248 | 100 | exp=1 | Reference |  |  |  |  |
|  |  |  | exp=2 | 4.7 | 3.57 | 6.2 | <0.001 |  |
|  |  |  | exp=3 | 3.82 | 2.89 | 5.04 | <0.001 |  |
|  |  |  |  |  |  |  |  |  |
| Age |  |  |  |  |  |  |  | 0.396 |
| <=60 | 1548 | 68.9 | exp=1 | Reference |  |  |  |  |
|  |  |  | exp=2 | 4.88 | 3.44 | 6.92 | <0.001 |  |
|  |  |  | exp=3 | 3.96 | 2.81 | 5.58 | <0.001 |  |
|  |  |  | exp=4 | 3.26 | 2.32 | 4.59 | <0.001 |  |
| >60 | 700 | 31.1 | exp=1 | Reference |  |  |  |  |
|  |  |  | exp=2 | 4.12 | 2.6 | 6.51 | <0.001 |  |
|  |  |  | exp=3 | 3.6 | 2.23 | 5.82 | <0.001 |  |
|  |  |  | exp=4 | 4.22 | 2.58 | 6.91 | <0.001 |  |
| Gender |  |  |  |  |  |  |  | 0.455 |
| Male | 1027 | 45.7 | exp=1 | Reference |  |  |  |  |
|  |  |  | exp=2 | 4.9 | 3.3 | 7.29 | <0.001 |  |
|  |  |  | exp=3 | 4.23 | 2.8 | 6.4 | <0.001 |  |
|  |  |  | exp=4 | 4.23 | 2.84 | 6.3 | <0.001 |  |
| Female | 1221 | 54.3 | exp=1 | Reference |  |  |  |  |
|  |  |  | exp=2 | 4.52 | 3.07 | 6.65 | <0.001 |  |
|  |  |  | exp=3 | 3.55 | 2.44 | 5.18 | <0.001 |  |
|  |  |  | exp=4 | 2.83 | 1.92 | 4.18 | <0.001 |  |
| Smoke |  |  |  |  |  |  |  | 0.263 |
| No | 1317 | 58.6 | exp=1 | Reference |  |  |  |  |
|  |  |  | exp=2 | 4.18 | 2.91 | 5.99 | <0.001 |  |
|  |  |  | exp=3 | 3.24 | 2.27 | 4.63 | <0.001 |  |
|  |  |  | exp=4 | 3.4 | 2.34 | 4.94 | <0.001 |  |
| Yes | 931 | 41.4 | exp=1 | Reference |  |  |  |  |
|  |  |  | exp=2 | 5.58 | 3.62 | 8.58 | <0.001 |  |
|  |  |  | exp=3 | 5.11 | 3.27 | 7.99 | <0.001 |  |
|  |  |  | exp=4 | 3.5 | 2.3 | 5.32 | <0.001 |  |

**Figure S1**. Flowchart of this study.
